# Supplementary material for: All-optical geometric image transformations enabled by ultrathin metasurfaces
Source: Nat Commun. 2023 Dec 15;14:8374. doi: 10.1038/s41467-023-43981-x (PMC10724155; doi:10.1038/s41467-023-43981-x)
Supplement: Supplementary file 1 — Supplementary Information [file 41467_2023_43981_MOESM1_ESM.pdf]

Supplementary Information for

All-optical geometric image transformations enabled by ultrathin metasurfaces

Xingwang Zhang[#], Xiaojie Zhang[#], Yao Duan, Lidan Zhang, and Xingjie Ni[*]

Department of Electrical Engineering, The Pennsylvania State University, University Park, PA, 16802, United States

[#] These authors contribute equally to this paper

* Email: xingjie@psu.edu

Contents

1. The theory for geometric image transformation using metasurfaces.....................3
2. The small angle approximation...8
3. The neighboring coupling effect in metasurfaces...10
4. The optimization for metasurface design..14
5. Sample fabrication 16
6. Experimental setup..18
7. Sampling rate analysis in log-polar coordinate transformation 19
8. More data on the scale- and rotation-invariance image transformations.............21
9. Log-polar to Cartesian coordinate transformation using metasurfaces 24
10. Metasurfaces for multi-color geometric image transformations..........................27
11. The effect of linear translation on the Cartesian to log-polar coordinate transformation 32
12. Supplementary References..40

1. The theory for geometric image transformation using metasurfaces

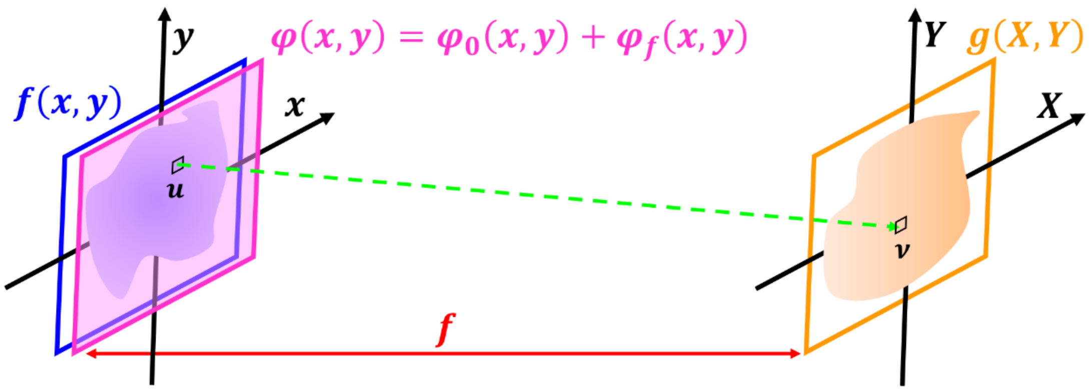

Figure S1. A schematic for field mapping from (*x*, *y*) plane to the focal plane (*X*, *Y*) of a metasurface. Under normal incidence, the light transmitted from each pixel of an image with amplitude-only transmittance $f(x, y)$ in the (*x*, *y*) plane is directed by the metasurface with a phase profile $\varphi(x, y)$ to (*X*, *Y*) plane, forming an image with a redistributed intensity profile $g(X, Y)$.

We assume an input image with an amplitude-only transmittance function $f(x, y)$ is projected onto a metasurface with a phase-only transmittance function $e^{i\varphi(x,y)}$, and then transformed to an image $g(X, Y)$. As illustrated in Fig. S1, the phase profile $\varphi(x, y)$ for the metasurface can be expressed as:

$$\varphi(x, y) = \varphi_0(x, y) + \varphi_f(x, y), \quad \text{(S1)}$$

where $\varphi_0(x, y)$ is the phase encoded by the desired geometric image transformation, and $\varphi_f(x, y)$ is the phase of a Fourier transform lens, and we have

$$\varphi_f(x, y) = -k \cdot \sqrt{x^2 + y^2 + f^2}, \quad \text{(S2)}$$

where, *f* is the focal length and *k* is the wave number. Under the paraxial approximation condition, the corresponding output image $g(X, Y)$ can be described by a modified

Fourier transform from the field on the metasurface plane (x, y) to that on the output plane (X, Y)

$$g(X,Y) = \iint f(x,y) \cdot e^{i\varphi_0(x,y)} \cdot e^{-ik(x \cdot X + y \cdot Y)/f} dxdy \tag{S3}$$

In the absence of $\varphi_0(x, y)$, the kernel of the integral becomes a Fourier kernel. In this scenario, the metasurface functions as a Fourier transform lens, and $g(X, Y)$ represents the spatial frequency spectrum of the input image $f(x, y)$. In the presence of $\varphi_0(x, y)$, the kernel of the integral turns into a complex term, with the phase being modulated by the additional spatially variant phase distribution $\varphi_0(x, y)$. As a result, the phase term $\varphi_0(x, y)$ incorporated in the Fourier transform metasurface lens can be utilized to geometrically transform the input image $f(x, y)$ into $g(X, Y)$. This can also be understood as follows: for normal light incidence on the metasurface with a phase profile of $\varphi_0(x, y)$, the local light deflection angle in the (x, z) plane can be expressed as

$$\sin\beta = \frac{1}{k} \cdot \frac{\partial\varphi_0(x,y)}{\partial x}. \tag{S4}$$

The light is subsequently modulated by the Fourier transform phase profile $\varphi_f(x, y)$ and mapped onto the spatial frequency domain (X, Y). Under small angle approximation,[1] we have

$$\sin\beta \approx \tan\beta = \frac{X(x,y)}{f}. \tag{S5}$$

Similarly, in the (y, z) plane, we have

$$\sin\beta = \frac{1}{k} \cdot \frac{\partial\varphi_0(x,y)}{\partial y} \approx \tan\beta = \frac{Y(x,y)}{f}. \tag{S6}$$

Therefore, we derived the phase gradient of $\varphi_0(x, y)$,

$$\frac{\partial\varphi_0(x,y)}{\partial x} = \frac{k}{f} \cdot X(x,y), \quad \text{(S7)}$$

$$\frac{\partial\varphi_0(x,y)}{\partial y} = \frac{k}{f} \cdot Y(x,y). \quad \text{(S8)}$$

We first consider only the geometric characteristics of the image $f(x,y)$ and neglected the grayscale information. In this case, the image $f(x,y)$ can be expressed as:

$$f(x,y) = \begin{cases} 1, & x,y \in \mathrm{A} \\ 0, & x,y \notin \mathrm{A}' \end{cases} \quad \text{(S9)}$$

where, A is the outline of the image. For the log-polar coordinate transform of an image, we have the coordinate relations as below:

$$X(x,y) = a \cdot \ln\frac{r}{b}, \quad \text{(S10)}$$

$$Y(x,y) = -a \cdot \alpha, \quad \text{(S11)}$$

$$r = \sqrt{x^2 + y^2}, \quad \text{(S12)}$$

$$\alpha = \mathrm{atan2}(y,x), \quad \text{(S13)}$$

where a and b are scale factors. In order to obtain the encoded phase term $\varphi_0(x,y)$ of metasurfaces for the log-polar coordinate transform, we substituted the coordinate transformation relations Eqs. S10-S13 into Eqs. S7-S8. By integrating the spatial phase gradient of $\varphi_0(x,y)$, we can then derive

$$\varphi_0(x,y) = \frac{k}{f} \cdot [x \cdot X(x,y) + y \cdot Y(x,y) - a \cdot x]. \quad \text{(S14)}$$

Therefore, combing Eq. S1, Eq. S2 and Eq. S14, we can obtain the phase profile $\varphi(x,y)$ of metasurfaces for log-polar coordinate transformation:

$$\varphi(x,y) = \frac{k}{f} \cdot [x \cdot X(x,y) + y \cdot Y(x,y) - a \cdot x] - k \cdot \sqrt{x^2 + y^2 + f^2}. \quad \text{(S15)}$$

For the log-polar coordinate transformation, we considered only the geometric characteristics of the image $f(x,y)$ and neglected the grayscale information. Therefore, the metasurfaces for log-polar coordinate transformation only works for binary images whose pixels consist of only two intensity values (Eq. S9). To transform

grayscale images with both grayscale and local geometric information, we need to incorporate both grayscale and geometric information of the image $f(x,y)$. To this end, we can divide both the input image $f(x,y)$ and the output image $g(X,Y)$ into N parts with an equal amount of energy. An arbitrary small piece u in the (x,y) plane is then redirected by the metasurface to v in (X,Y) plane (Fig. S1). Based on the energy conservation law, the energy in u and v should be equal, resulting in $\int_u f(x,y)\,du = \int_v g(X,Y)\,dv$.[2] Therefore, for the mapping from (x,y) to (X,Y), we have the expression

$$\iint_{x,y=0}^{x,y} f(x,y)\,dx\,dy = \iint_{X,Y=0}^{X,Y} g(X,Y)\,dX\,dY. \tag{S16}$$

As an example, we considered a transformation from a Gaussian grayscale profile to a square flat-top profile:

$$f(x,y) = \exp\left[-\frac{2(x^2+y^2)}{r_0^2}\right], \tag{S17}$$

$$g(X,Y) = \frac{1}{4w_0^2}\cdot \mathrm{rect}\left(\frac{X}{2w_0}\right)\cdot \mathrm{rect}\left(\frac{Y}{2w_0}\right), \tag{S18}$$

where r_0 is the radius of the Gaussian profile and w_0 is the half of the width of the flat-top profile. By substituting Eqs. S17 and S18 into Eq. S16, we can derive the coordinate transformation relationships:

$$X = w_0\cdot \mathrm{erf}\left(\sqrt{2}\frac{|x|}{r_0}\right), \tag{S19}$$

$$Y = w_0\cdot \mathrm{erf}\left(\sqrt{2}\frac{|y|}{r_0}\right), \tag{S20}$$

where $\mathrm{erf}(\xi)$ is error function and is defined as $\mathrm{erf}(\xi) = \frac{2}{\sqrt{\pi}}\int_0^{\xi}\exp(-\xi^2)\,d\xi$. Combing the Eqs. S19-S20 with Eqs. S7 and S8, we can finally obtain the required transformation phase profile by integrating the spatial phase gradient of $\varphi_0(x,y)$, leading to

$$\varphi_0(x,y) = 2\sqrt{2\pi}\frac{r_0 w_0}{f\lambda}\cdot\left\{\left[\frac{\sqrt{\pi}}{2}\cdot\xi_x\cdot\mathrm{erf}(\xi_x)+\frac{1}{2}\cdot\exp\left(-{\xi_x}^2\right)-\frac{1}{2}\right]+\left[\frac{\sqrt{\pi}}{2}\cdot\xi_y\cdot\mathrm{erf}(\xi_y)+\frac{1}{2}\cdot\exp\left(-{\xi_y}^2\right)-\frac{1}{2}\right]\right\},$$

(S21)

where $\xi_x = \sqrt{2}\frac{|x|}{r_0}$ and $\xi_y = \sqrt{2}\frac{|y|}{r_0}$. Therefore, combing Eq S1, Eq. S2 and Eq. S21, we can obtain the phase profile $\varphi(x, y)$ of metasurfaces for Gaussian-to-flat-top transformation:

$$\varphi(x, y) = 2\sqrt{2\pi}\frac{r_0 w_0}{f\lambda} \cdot \left\{\left[\frac{\sqrt{\pi}}{2} \cdot \xi_x \cdot \mathrm{erf}(\xi_x) + \frac{1}{2} \cdot \exp\left(-{\xi_x}^2\right) - \frac{1}{2}\right] + \left[\frac{\sqrt{\pi}}{2} \cdot \xi_y \cdot \mathrm{erf}\left(\xi_y\right) + \frac{1}{2} \cdot \exp\left(-{\xi_y}^2\right) - \frac{1}{2}\right]\right\} - k \cdot \sqrt{x^2 + y^2 + f^2}.$$

(S22)

In this work, the phase profiles used for Cartesian to log-polar and Gaussian-to-flat-top transformations are plotted in Fig. S2.

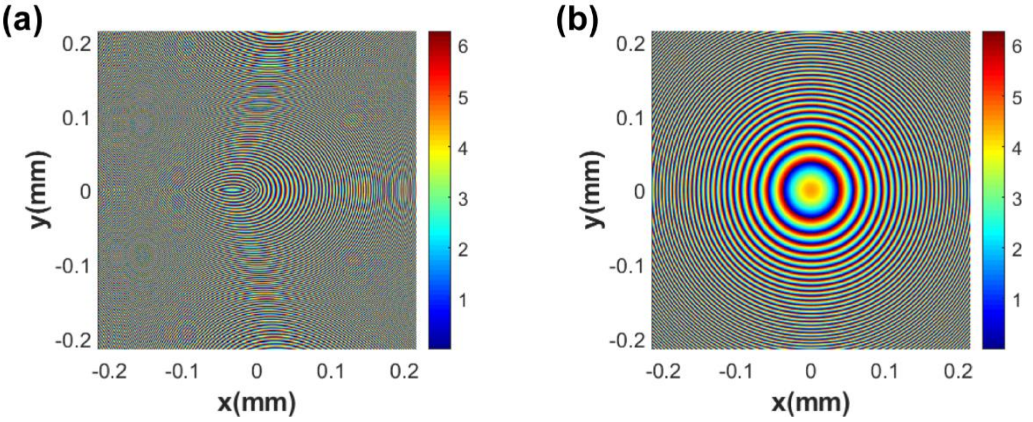

Figure S2. The phase profiles for Cartesian to log-polar (a) and Gaussian-to-flat-top (b) transformations.

2. The small angle approximation

In Supplementary Note 1, we used small angle approximation to simplify the derivation and obtain $\sin\beta \approx \tan\beta = \frac{X(x,y)}{f}$ in the (x,z) plane and $\sin\beta \approx \tan\beta = \frac{Y(x,y)}{f}$ in the (y,z) plane. In this section, we evaluated the accuracy for the small angle approximation and the effect on the geometric image transformation.

Taking the log-polar coordinate transformation as an example. Combing Eqs. S5 and S10, we can derive $\sin\beta \approx \tan\beta = \frac{a}{f} \cdot \ln\frac{r}{b}$ in the (x,z) plane. Similarly, we have $\sin\beta \approx \tan\beta = \frac{-a \cdot \alpha}{f}$ in the (y,z) plane. In our work, $a = 30\ \mu m$, $b = 100\ \mu m$, $f = 400\ \mu m$ and $\alpha \in (-\pi, \pi)$. In the (y,z) plane, we have $\tan\beta \in (-0.2356, 0.2356)$ and the angle range $\beta \in (-0.2314, 0.2314)$. For such an angle range, the range for $\sin\beta$ is $(-0.2293, 0.2293)$ which yields a maximum error of 0.92%. In the (x,z) plane, the angle range of β and the error are proportional to r (i.e. the size of image). The images we used in this work are all below 100 μm, corresponding to an angle of 0.0137 rad and an error of 0.94%.

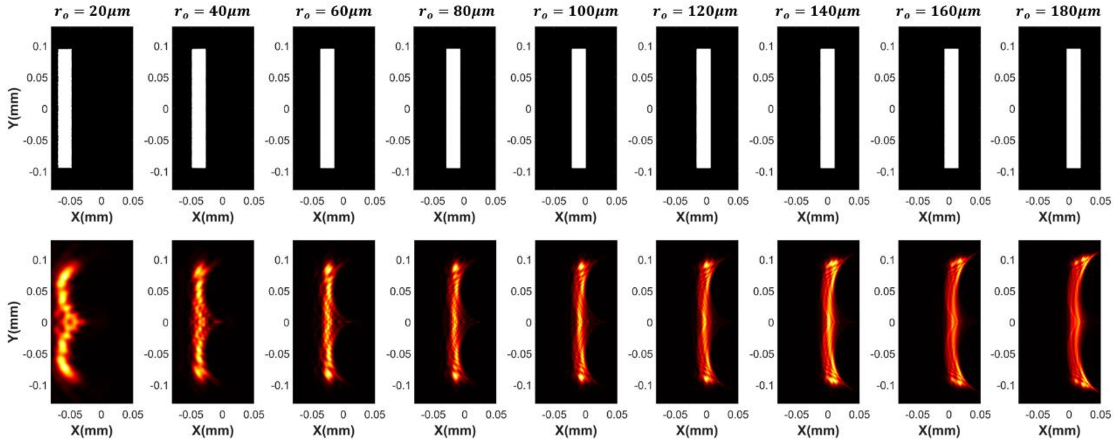

Figure S3. The log-polar coordinate transformation for different size of ring-shaped images. Upper: theoretical calculation results. Bottom: simulation results. r_o is the outer radius for the ring shapes, and the inner radius is fixed as a half of the outer radius**.**

To better understand the effect of image size on the geometric image transformation, we simulated the log-polar transformation for different sizes of ring-shaped images and compared with theoretical prediction. As can be seen in Fig. S3, the transformed images have a relatively smaller distortion when the outer radius r_o of the ring shapes between 40 μm and 100 μm. For the rings with $r_o > 100\ \mu m$, due to the increased error for the small angle approximation, the transformed images are distorted. For the ring-shaped image with r_o=20 μm, the effective numerical aperture for the metasurfaces $\mathrm{NA} = r_o/f = 0.05$, which yields a spatial resolution of 13 μm at the operation wavelength $\lambda = 1.064\ \mu m$ Consequently, due to the spatial resolution limitation, the transformed image for r_o=20 μm is also distorted. Therefore, our metasurfaces can only operate for the geometric transformation of images with the size in the range between 40 μm and 100 μm with negligible aberration.

3. The neighboring coupling effect in metasurfaces

To evaluate the effects of meta-atom neighboring coupling on optical geometric transformations, we first utilized conventional nanodisk-based meta-atoms to build metasurfaces. As can be seen in Fig. S4a, a unit cell of the metasurface consists of an amorphous silicon nanodisk sitting on fused silica substrate. We then calculated the phase shift and transmittance of a square lattice of nanodisks for normal light incidence as a function of duty cycle (i.e. diameter/period) by assuming the neighboring nanodisks were identical. As shown in Fig. S4b, the phase shift can fully cover 2π with uniform high transmittance by varying the diameter of the silicon nanodisk.

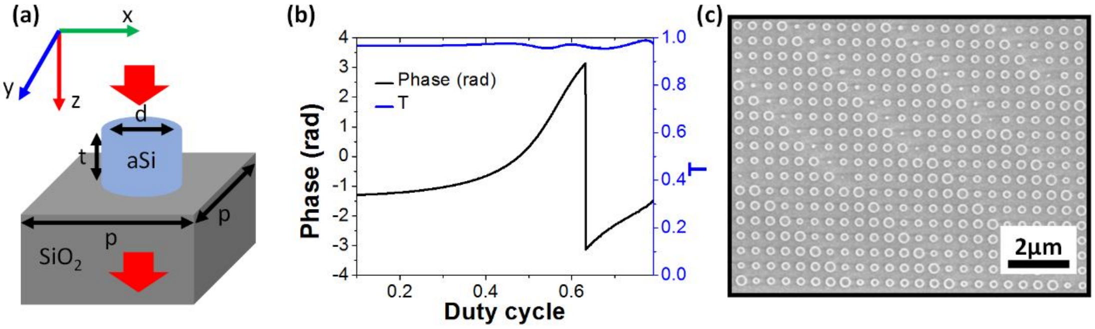

Figure S4. Design of metasurfaces based on nanodisk array. (a) Schematic of a unit cell of metasurfaces consisting of an amorphous silicon nanodisk on fused silica substrate. The period of meta-atoms is p. The thickness and diameter of the nanodisk are represented by t and d, respectively. (**b)** The phase shift and transmittance of the nanodisk array imparted on the incidence light as a function of duty cycle (i.e. diameter/period). **(c)** A representative scanning electron microscopy (SEM) image of metasurfaces based on amorphous silicon nanodisk array.

However, the neighboring nanodisks in real metasurfaces have different dimensions, which may introduce additional phase discrepancy on the incidence light due to the neighboring coupling between different sized nanodisks (Fig. S4c). To evaluate the effects of neighboring coupling on geometric transformations, we took the Gaussian-to-flat-top transformation as an example. To this end, we calculated the

optical field propagation without considering the neighboring coupling effect. As shown in Fig. S5a and 5b, the input Gaussian beam with a radius of 6 μm at z=0 mm smoothly turned into a uniform flat-top beam with a width of 12 μm. To incorporate the neighboring coupling between meta-atoms, we conducted full wave simulation using Finite-difference time-domain (FDTD) method. In contrast to the uniform optical field transition in Fig. S5a, we observed wave-like intensity distribution in the beam propagation plane, and the transformed flat-top beam exhibited significant intensity fluctuations (Figs. S5c-S5d). Therefore, the neighboring coupling between meta-atoms can severely affect the optical geometric transformation when nanodisks are used as meta-atoms.

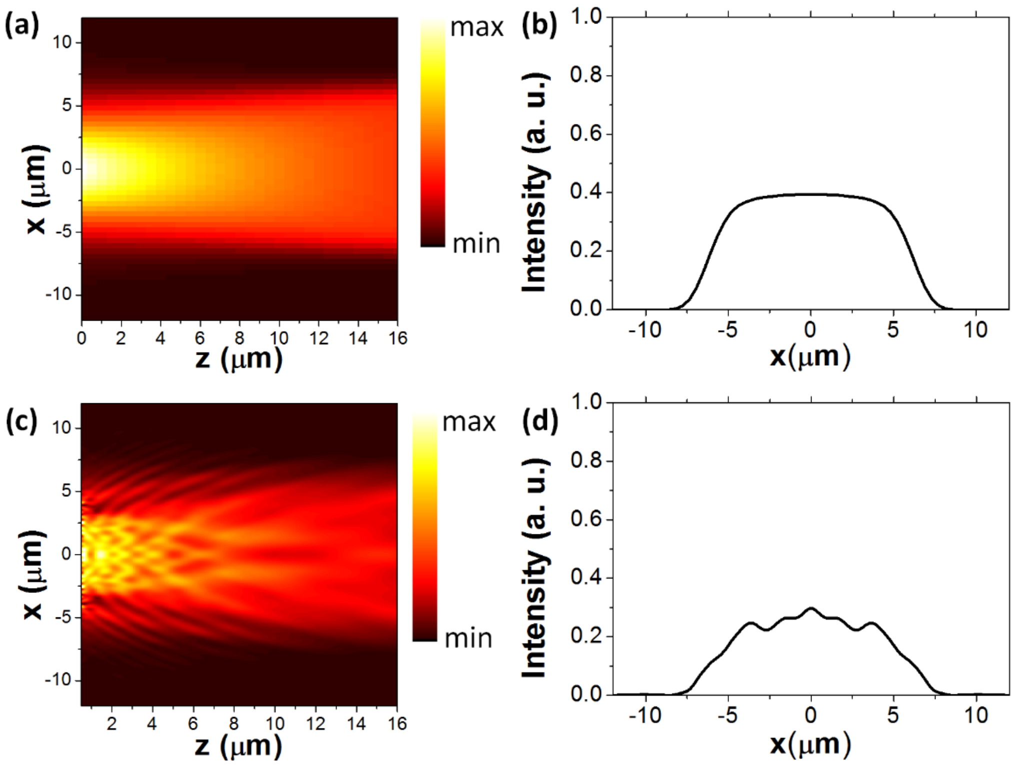

Figure S5. Effects of neighboring coupling on optical geometric transformations. The analytical **(a)** and Finite-difference time-domain (FDTD) **(c)** calculation results for the intensity distribution when a Gaussian beam with a radius of 6 μm propagates along z axis. The metasurface was placed at z=0 μm and the flat-top beam was expected to be formed at z=14 μm. The transformed flat-top beam profile at z=14 μm for analytical **(b)** and FDTD **(d)** calculation.

Having evaluated the effects of neighboring coupling of nanodisks on the performance of optical geometric transformation using metasurfaces, we continued to study the dimension tolerance of nanodisks. In this respect, we increased the radius of all the nanodisks by 10 nm. According to our simulations, the converted flat-top beam turned out to be extremely distorted (Fig. S6). Therefore, the geometric transform metasurfaces designed by nanodisks are extremely sensitive to the fabrication imperfection.

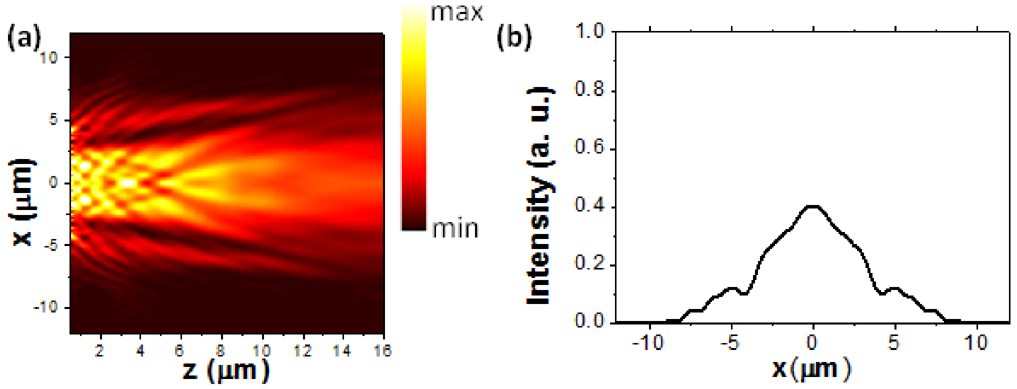

Figure S6. The effects of fabrication imperfection effects on optical geometric transformations. (a) The FDTD simulation results for the intensity distribution when a Gaussian beam with a radius of 6 µm propagating along z axis is transformed to flat-top beam by a metasurface consisting of nanodisks. **(b)** The transformed flat-top beam profile at z=14 µm.

To experimentally evaluate the fabrication tolerance and neighboring coupling effects, we fabricated nanodisks based metasurfaces for Gaussian-to-flat-top transformation. As can be seen in Fig. S7, compared with the perfect theoretical prediction, the transformed flat-top beam profile is severely distorted due to the inevitable fabrication imperfection and neighboring coupling effects.

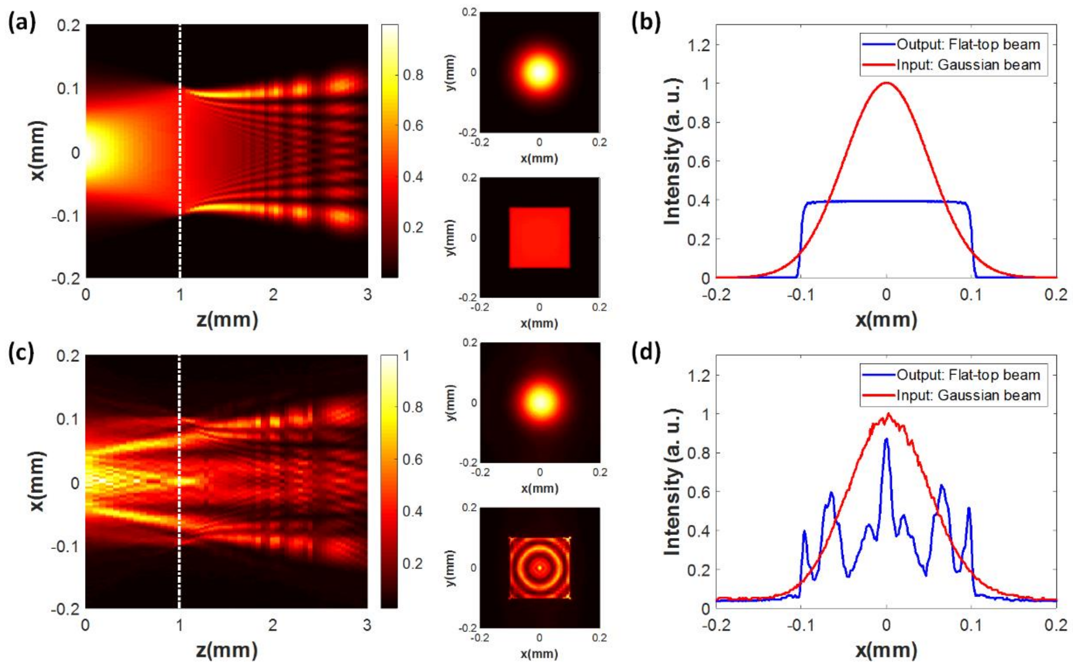

Figure S7. The analytical **(a-b)** and experimental **(c-d)** demonstration for the transformation of a Gaussian beam with a radius of 100 μm (upper right inset in **(a)** and **(c)**) to a flat-top beam with a width of 200 μm (lower right inset in **(a)** and **(c)**). The metasurfaces are designed by nanodisks in **Fig. S4**. **(b)** and **(d)** are the analytical and experimental results for the beam profiles at z=14 μm.

4. The optimization for metasurface design

To evaluate the performance of our optimized metasurface design in Fig. 2 in the main text, we first used our selected meta-atom (lx=330 nm, ly=140 nm, labeled by a star in Fig. 2c in the main text) to construct a metasurface for Gaussian-to-flat-top transformation, and the spatial arrangement of meta-atoms followed the linear dependence of phase on orientation angle. As can be seen in Fig. S8a, the output beam profile is close to a flat-top shape, indicating much less neighboring coupling effects than that of the nanodisk meta-atoms in Fig. S7d. We also calculated the output flat-top beam profile using a meta-atom with lx=350 nm and ly=170 nm (labeled by a diamond in Fig. 2c in the main text) as a comparison. This meta-atom has the same conversion efficiency (η) as the selected one but with a larger phase variance (σ^2) (Fig. 2c in main text). Consequently, the output flat-top beam profile for this metasurface is severely distorted as shown in Fig. S8b. Therefore, our selected meta-atom with lx=330 nm and ly=140 nm has the minimized neighboring coupling effects.

To further reduce the effects of neighboring coupling, we spatially arranged meta-atoms in the metasurface by following the nonlinear dependence of phase on orientation angles as shown in Fig. 2d in the main text. Figure S8c reveals that the output flat-top beam profile for the meta-atoms arranged by nonlinear phase dependence exhibits a better flatness than that in Fig. S8a, indicating weaker neighboring coupling effects.

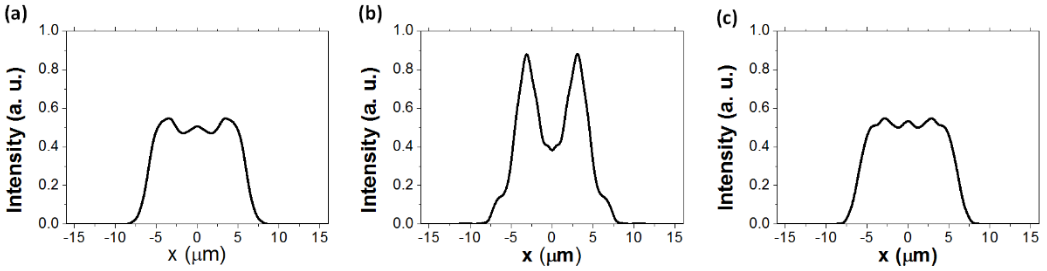

Figure S8. Evaluation of the performance of optimized metasurfaces. (a) The transformed flat-top beam profile by using the selected meta-atom (lx=330 nm, ly=140 nm, labeled by a star in Fig. 2c in the main text) with the meta-atom spatial arrangement following the linear dependence of phase on orientation angle. **(b)** The transformed flat-top beam profile by metasurfaces by using a meta-atom (lx=360 nm, ly=170 nm,

labeled by a diamond in Fig. 2c in the main text) which has the same conversion efficiency as the selected meta-atom (lx=330 nm, ly=140 nm) but higher neighboring coupling effects. **(c)** The transformed flat-top beam profile by using the selected meta-atom (lx=330 nm, ly=140 nm) with the meta-atom spatial arrangement following the nonlinear dependence of phase on orientation angle as shown in Fig. 2d in the main text to further minimize the neighboring coupling effects.

5. Sample fabrication

To fabricate metasurfaces for geometric image transformation, we first deposited a layer of amorphous silicon film with a thickness of 500 nm on a fused silica substrate by Plasma Enhanced Chemical Vapor Deposition (PECVD). Then we spin-coated e-beam resist on top of the silicon film followed by e-beam lithography. After development, we deposited a thin layer of Aluminum on the sample as the hard mask by e-beam evaporation for the dry etching followed by a lift-off process. Then we transferred the pattern onto the silicon layer by using Inductively Coupled Plasma - Reactive Ion Etching (ICP-RIE). Finally, we removed the residual Aluminum layer by wet-etching method. Figure S9 shows the optical microscopy image and the scanning electron microscopy (SEM) image for a representative fabricated metasurface for geometric image transformation.

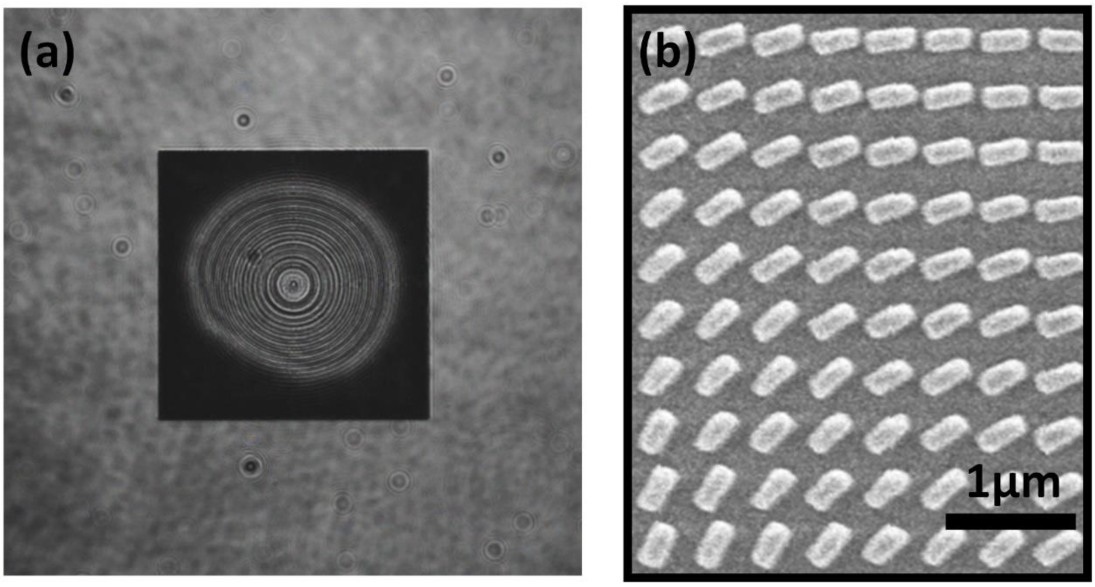

Figure S9. Optical microscopy image (a) and scanning electron microscopy (SEM) image (b) for a representative fabricated metasurface for geometric image transformation.

To fabricate the test images for log-polar coordinate transformation, we first deposited a layer of Aluminum film with a thickness of 100 nm on a glass slide by e-beam evaporation. The transmittance for 100 nm-thick Aluminum film is about 10^{-6} around $\lambda = 1064$ nm, which is small enough to block the incident laser beam. Then we spin-coated a layer of photo-resist on top of Aluminum film. After that, we used a laser writer to expose the patterns of the test images on the photo-resist. After development, we transferred the pattern onto the Aluminum film by using Inductively Coupled Plasma - Reactive Ion Etching (ICP-RIE). As such, the transparent region of the patterned Aluminum film formed the test images for log-polar coordinate transformation (Fig. S10).

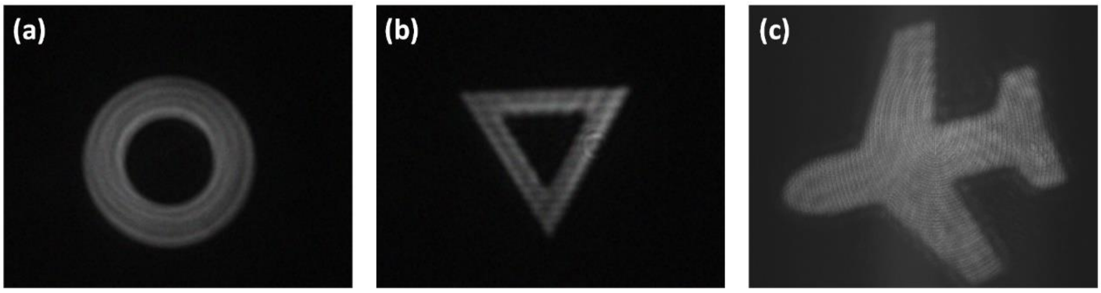

Figure S10. Optical microscopy images for the test images fabricated by a patterned Aluminum coated glass slide. The bright regions are transparent glass and the dark regions are 100nm-thick Aluminum coated glass. **(a)** A ring-shaped image; **(b)** A triangle-shaped image; **(c)** An airplane-shaped image.

6. Experimental setup

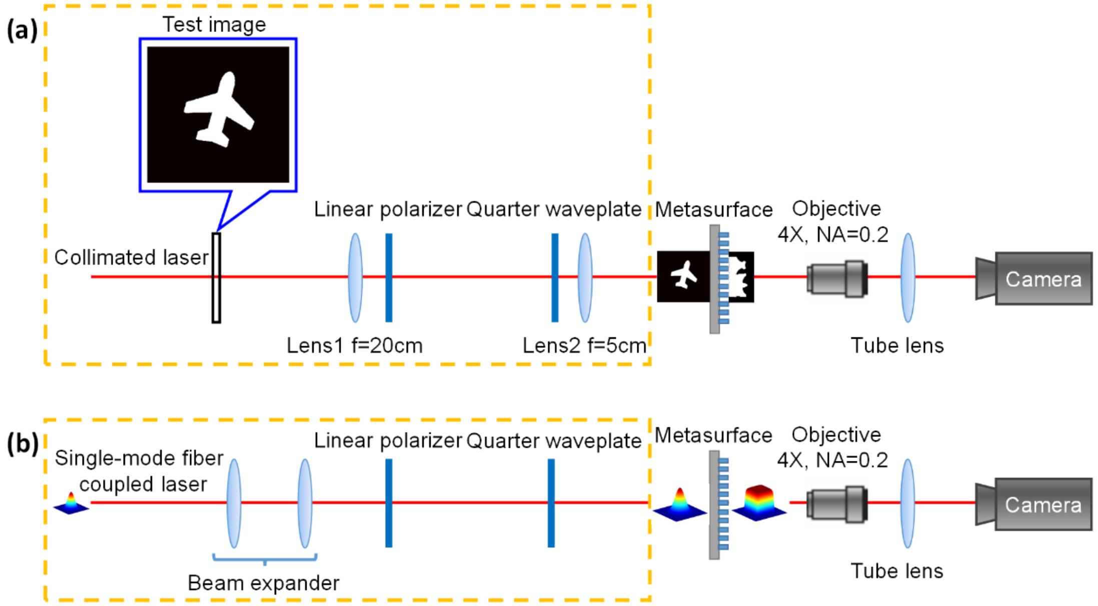

Figure S11. Schematic of the optical setup used for characterization. (a) The experiment setup for binary image transformation. **(b)** The experiment setup for grayscale image transformation.

In our experiment, we utilized a collimated laser beam (λ=1064 nm) as the light source for illumination and used a linear polarizer and a quarter wave plate to prepare a circular polarization state to meet the requirement of the metasurface. We then used a 4× objective (NA=0.2) and a tube lens to project the transformed images on a camera. For the binary image transformation, the collimated laser illuminated the patterned Aluminum coated glass slide to prepared the test images which were scaled down by 4× by a 4-f system and projected on the metasurface plane (Fig. S11a, Supplementary Note 3).

For the grayscale image transformation, the laser was coupled by a single-mode fiber to ensure single-mode Gaussian beam output. The Gaussian beam was then expanded by a beam expander and projected on the metasurface plane (Fig. S11b).

7. Sampling rate analysis in log-polar coordinate transformation

According to the log-polar coordinate transformation relationship, an image at the Cartesian coordinate (x,y) is projected to the log-polar coordinate (X,Y) by following $X(x,y) = a \cdot \ln\frac{r}{b}$ and $Y(x,y) = -a \cdot [\mathrm{atan2}(y,x)]$, in which $a = 30\ \mu m$ and $b = 100\ \mu m$ in this work. Clearly, when we sample the image with uniform sampling rate in the (x,y) domain, the transformed image in the (X,Y) domain will exhibits uneven sampling accordingly. To show such sampling rate transformation, we analyzed the log-polar coordinate transformation of a binary triangle-shaped object. As can be seen in Fig. S12a, the triangle was sampled with square lattice uniformly in (x,y) domain. After log-polar coordinate transformation, the sampling rate at the corners which are far from the origin becomes larger than the other regions ($\Delta X = a\frac{\Delta r}{r}$) (Fig. S12b). In our experiment, the image was uniformly illuminated and the meta-atoms array was arranged by a square lattice, both of which determined the uniform spatial sampling rate in the (x,y) domain. Therefore, after the image was transformed by the metasurface and projected onto CCD, the corner region with higher sampling rate became brighter.

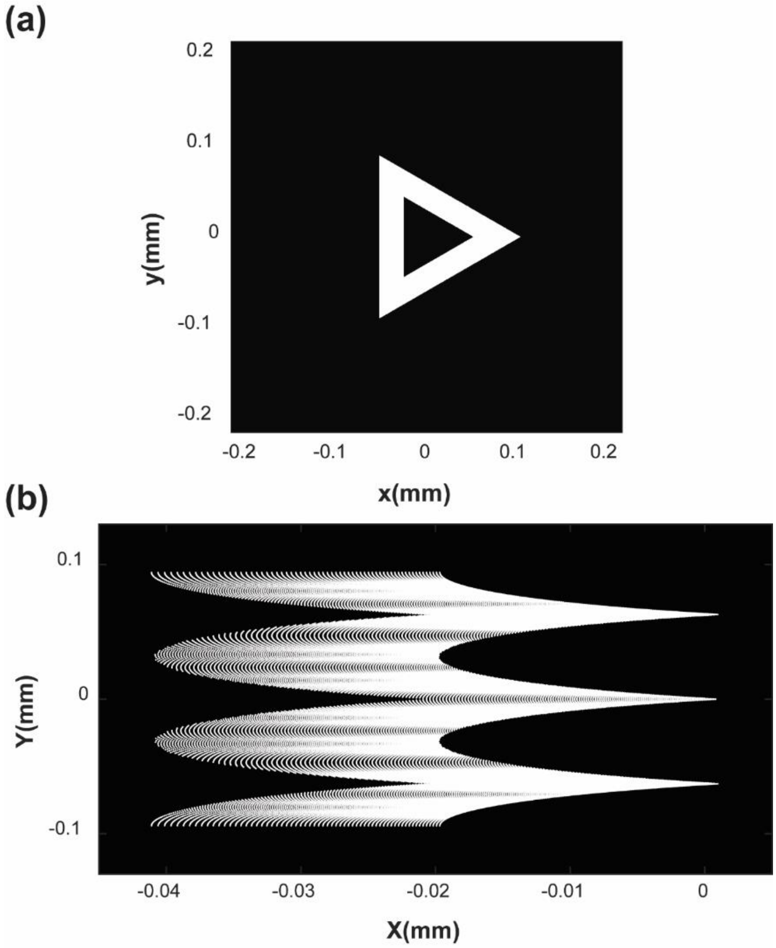

Figure S12. The image of a binary triangle-shaped object in the Cartesian coordinate (x,y) **(a)** and its log-polar coordinate transformation in the log-polar coordinate (X, Y) **(b)**, in which $X(x, y) = a \cdot \ln\frac{r}{b}$ and $Y(x, y) = -a \cdot [\mathrm{atan2}(y, x)]$ ($a = 30\ \mu m$ and $b = 100\ \mu m$). The transformation is conducted by mathematical calculation. In the Cartesian coordinate the target image is uniformly sampled, while in the log-polar coordinate the transformed image exhibits uneven sampling.

8. More data on the scale- and rotation-invariance image transformations

We chose triangle shapes which have distinct angular asymmetry as the test images to verify the scale- and rotation-invariance image transformation for our metasurfaces. We first used three triangles with the same orientation but different scaling factors (s = 1, 1.5, and 2.25) (Figs. S13a-c). According to the transformation relation, for the images with different scaling factors, the transformed images are expected to be invariant except for translations along the X axis ($X' = X + a \cdot \ln s$). To validate our theory, we conducted both numerical simulations and experiments using metasurfaces to process the three triangles. We observed three equally spaced peaks in all the transformed images, corresponding to three angles in the triangle shapes (Figs. S13d-i). The three peaks also have larger brightness due to higher sampling rate in the log-polar coordinate. Furthermore, all those transformed images are the same except different translations along the X axis indicating the size difference, which reveals that the log-polar image transform possesses scale-invariant property.

Having demonstrated the scale-invariant image transformation using metasurfaces, we continued to verify the rotation-invariant property. In this case, we utilized three triangles with the same size but different orientation angles (α = 0, -π/6, and -π/3) (Figs. S14a-14c), and the corresponding transformed images are expected to be the same except for the translations along the Y axis in the output plane ($Y' = Y - a \cdot \alpha$). To verify our prediction, we again conducted both numerical simulations and experiments

with our metasurfaces to process the three triangles. We observed that the transformed images agreed well with our prediction, which confirmed that the log-polar image transform possesses rotation-invariant property (Figs. S14d-14i).

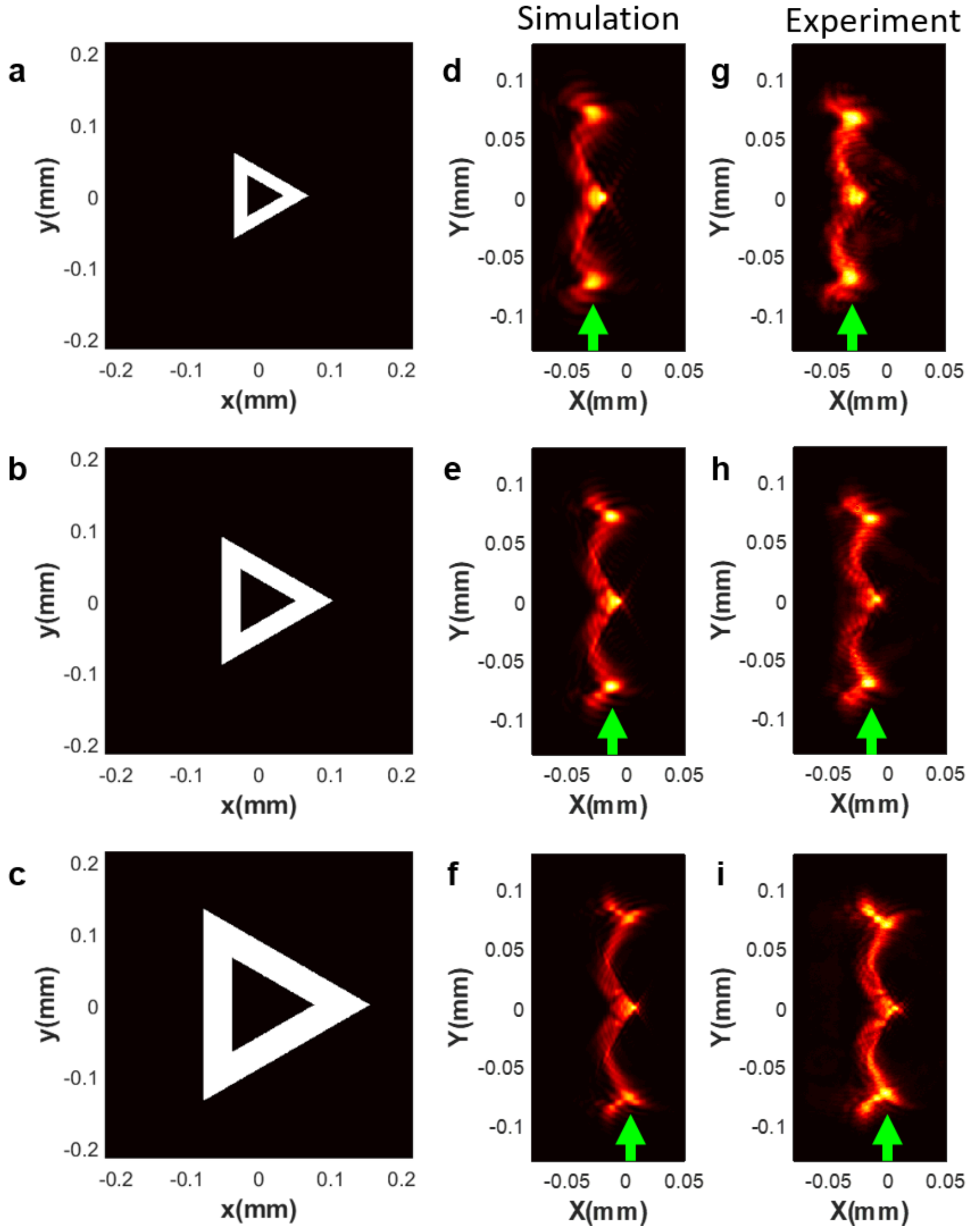

Figure S13. The scaling invariance of the log-polar coordinate transformation using metasurfaces. The triangle-shaped objects in the Cartesian coordinate with scaling factors (s) of s=1 (**a**), s=1.5 (**b**) and s=2.25 (**c**). The white and black regions indicate the transmittance of 100% and 0%, respectively. The simulation (**d-f**) and

measurement (**g-i**) images in the log-polar coordinate for the input images (**a-c**). The green arrows are used for eye guidance of the peak positions.

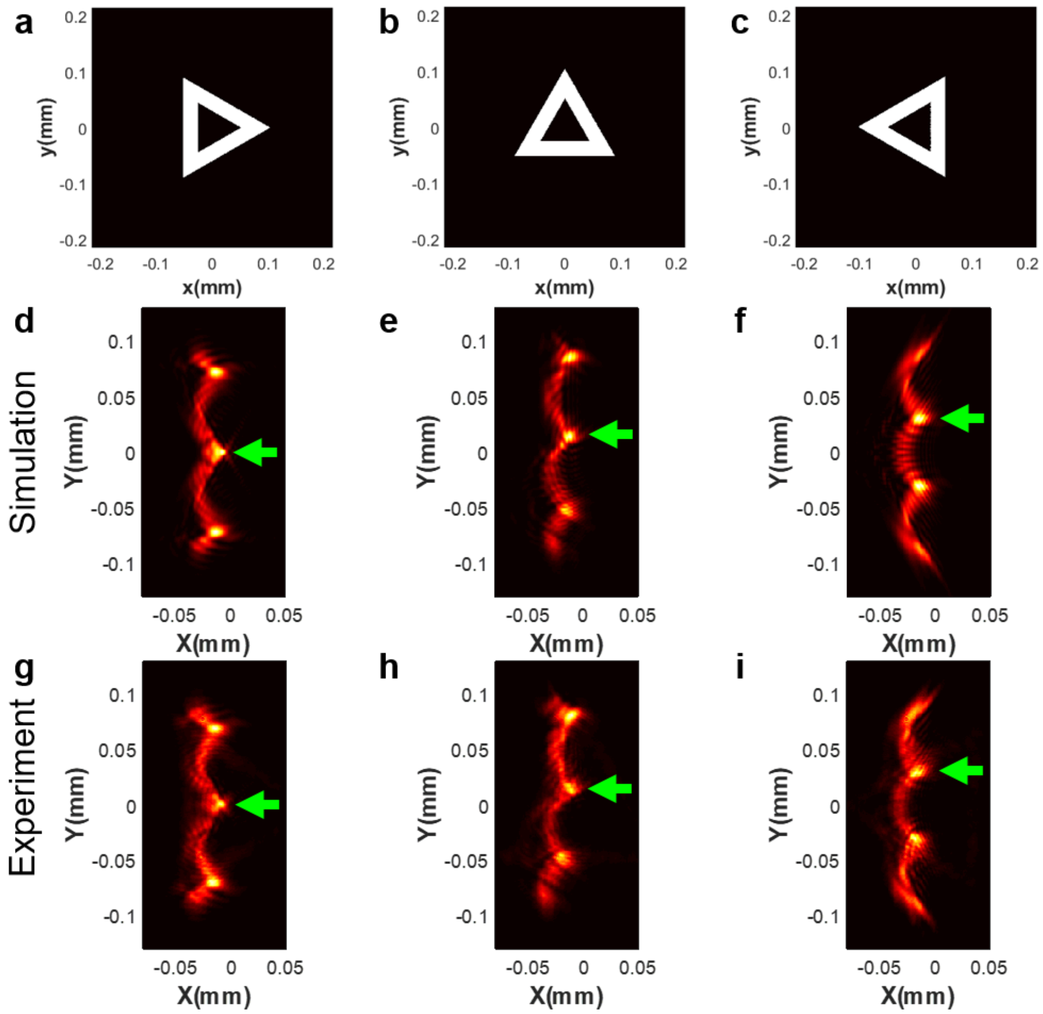

Figure S14. The rotation invariance of the log-polar coordinate transformation using metasurfaces. The triangle-shaped objects in the Cartesian coordinate with rotation angles (α) of α=0 (**a**), α=-π/6 (**b**) and α=-π/3 (**c**). The white and black regions indicate the transmittance of 100% and 0%, respectively. The simulation (**d-f**) and measurement (**g-i**) images in log-polar coordinate for the input images (**a-c**). The green arrows are used for eye guidance of the central peak positions.

9. Log-polar to Cartesian coordinate transformation using metasurfaces

In the main text, we have realized the image transformation from Cartesian to log-polar coordinate using metasurfaces. We can also utilize metasurfaces to perform log-polar to Cartesian coordinate transform of an image. In this case, we have a log-polar coordinate in the (x, y) plane:

$$x = a \cdot \ln\frac{r}{b}, \tag{S23}$$

$$y = -a \cdot \alpha. \tag{S24}$$

In the (X, Y) plane, we have the coordinate relations as below:

$$X(x, y) = r \cdot \cos(\alpha), \tag{S25}$$

$$Y(x, y) = r \cdot \sin(\alpha), \tag{S26}$$

$$r = b \cdot e^{\frac{x}{a}}, \tag{S27}$$

$$\alpha = -\frac{y}{a}, \tag{S28}$$

In order to obtain the encoded phase term $\varphi_0(x, y)$ of metasurfaces for the log-polar to Cartesian coordinate transform, we substituted the coordinate transformation relations Eqs. S25-S28 into Eqs. S7-S8. By integrating the spatial phase gradient of $\varphi_0(x, y)$, we can then derive

$$\varphi_0(x, y) = \frac{k}{f} \cdot a \cdot X(x, y). \tag{S29}$$

Therefore, combing Eq. S1, Eq. S2 and Eq. S29, we can obtain the phase profile $\varphi(x, y)$ of metasurfaces for log-polar to Cartesian coordinate transformation:

$$\varphi(x, y) = \frac{k}{f} \cdot a \cdot X(x, y) - k \cdot \sqrt{x^2 + y^2 + f^2}. \tag{S30}$$

For the log-polar to Cartesian coordinate transformation, we considered only the geometric characteristics of the image $f(x, y)$ and neglected the grayscale information. Therefore, the metasurfaces for log-polar to Cartesian coordinate

transformation only works for binary images whose pixels consist of only two intensity values (Eq. S9).

As we have demonstrated in Fig. 3 in the main text, the Cartesian to log-polar coordinate transformation for a ring is a rectangle. Conversely, for a rectangle in the log-polar coordinate, the transformed image in the Cartesian coordinate should be a ring. With this in mind, we projected a rectangle-shaped binary image as shown in Fig. S15a on a metasurface which was encoded by the phase profile in Eq. S30 to perform log-polar to Cartesian coordinate transformation. The rectangle image in Fig. S15a has the same size and location as the one in Fig. 3b in the main text, which was previously transformed by a ring with the inner radius of 50 μm and outer radius of 100 μm from Cartesian to log-polar coordinate. As can be seen in Fig. S15c, the transformed image in Cartesian coordinate is a ring which agrees well with the theoretical calculation result in Fig. S15b.

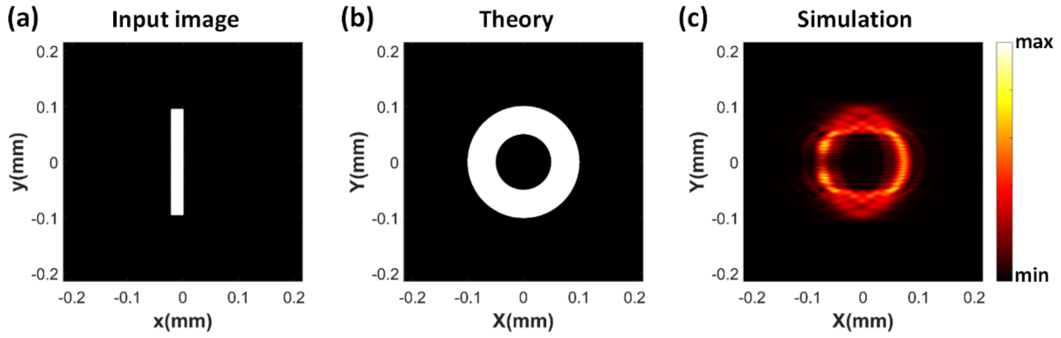

Figure S15. The log-polar to Cartesian coordinate transformation using metasurfaces to reconstruct the ring-shaped image in Fig. 3 in the main text.

On the other hand, we have transformed airplane-shaped images in Cartesian coordinate to log-polar coordinate using metasurfaces as shown in Fig. 4 in the main text. We can also utilize metasurfaces to transform the images in Figs. 4d-4f back to Figs. 4a-c by performing log-polar to Cartesian coordinate transformation. To this end, we projected the image in Fig. S16a on a metasurface which was encoded by the phase

profile in Eq. S30 to perform log-polar to Cartesian coordinate transformation. The image in Fig. S16a has the same shape and location as the one in Fig. 4e in the main text, which was previously transformed by the airplane-shaped image in Fig. 4b from Cartesian to log-polar coordinate. As can be seen in Fig. S16c, although the transformed image in Cartesian coordinate has rough intensity distribution due to the uneven sampling rate during the transformation, it has the same shape as the theoretical calculation result in Fig. S16b.

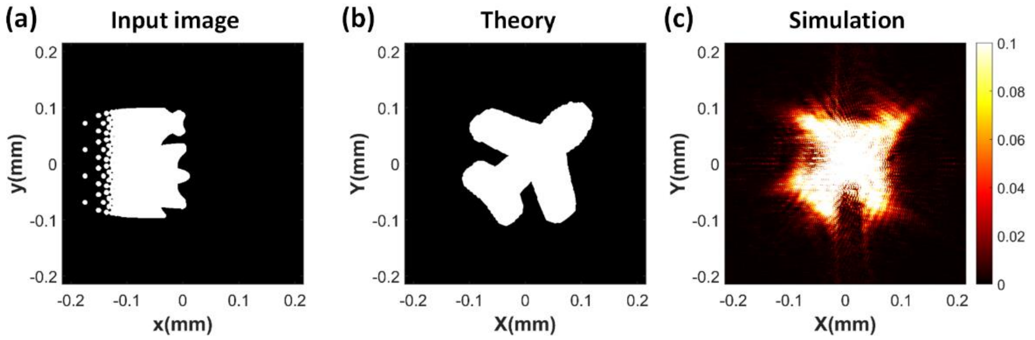

Figure S16. The log-polar to Cartesian coordinate transformation using metasurfaces to reconstruct airplane images in Fig. 4 in the main text.

10. Metasurfaces for multi-color geometric image transformations

From Eq. S15, we know the phase profile $\varphi(x, y)$ of metasurfaces for log-polar coordinate transformation is wavelength dependent. Therefore, we can re-write it as

$$\varphi(x, y, \lambda) = \frac{2\pi}{\lambda} \cdot \left\{\frac{1}{f} \cdot [x \cdot X(x, y) + y \cdot Y(x, y) - a \cdot x] - \sqrt{x^2 + y^2 + f^2}\right\}. \quad \text{(S31)}$$

For simplicity, we can obtain

$$\varphi(x, y, \lambda) = \frac{2\pi}{\lambda} \cdot \Phi(x, y), \quad \text{(S32)}$$

$$\Phi(x, y) = \frac{1}{f} \cdot [x \cdot X(x, y) + y \cdot Y(x, y) - a \cdot x] - \sqrt{x^2 + y^2 + f^2}, \quad \text{(S33)}$$

where $\Phi(x, y)$ is wavelength independent. In our work, the metasurfaces were design at $\lambda = 1064\,nm$. Therefore, if we change the wavelength of the incident laser, the transformed image will be distorted as a result of the chromatic phase aberration (Eq. S32).

On the other hand, the transmittance (i.e. LCP-to-RCP conversion efficiency) for our meta-atom was also wavelength dependent. As shown in Fig. S17, our meta-atom has a peak transmittance of about 98% at λ=1064 nm. When the wavelength deviates from λ=1064 nm, the transmittance drops down to about 60% at λ=980 nm and λ=1200 nm. Therefore, if we change the wavelength of the incident laser, the transformed image will become dim due to smaller transmittance efficiency.

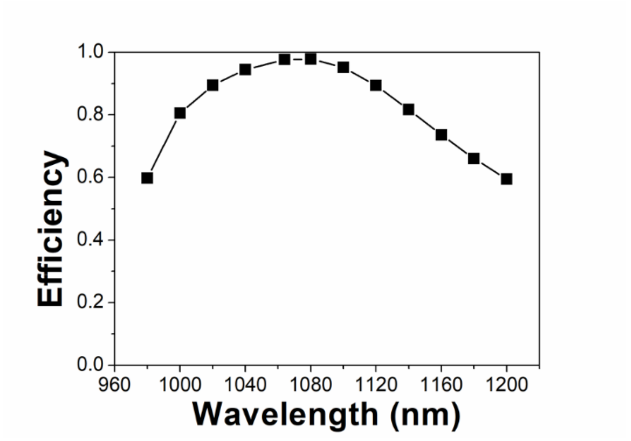

Figure S17. LCP-to-RCP conversion efficiency for our meta-atoms at different wavelengths.

To further study the wavelength dependent characteristics for our log-polar coordinate transformation, we simulated the transformation for the ring-shaped image in Fig. 3a in the main text at different wavelengths, and the results are plotted in Fig. S18. When the wavelength deviates from λ=1064 nm, the transformed images turn dim due to smaller transmittance. In addition, when the wavelength is beyond $\pm$20 nm away from λ=1064 nm, the transformed images become bended rectangles due to the chromatic aberration (Eq. S32)

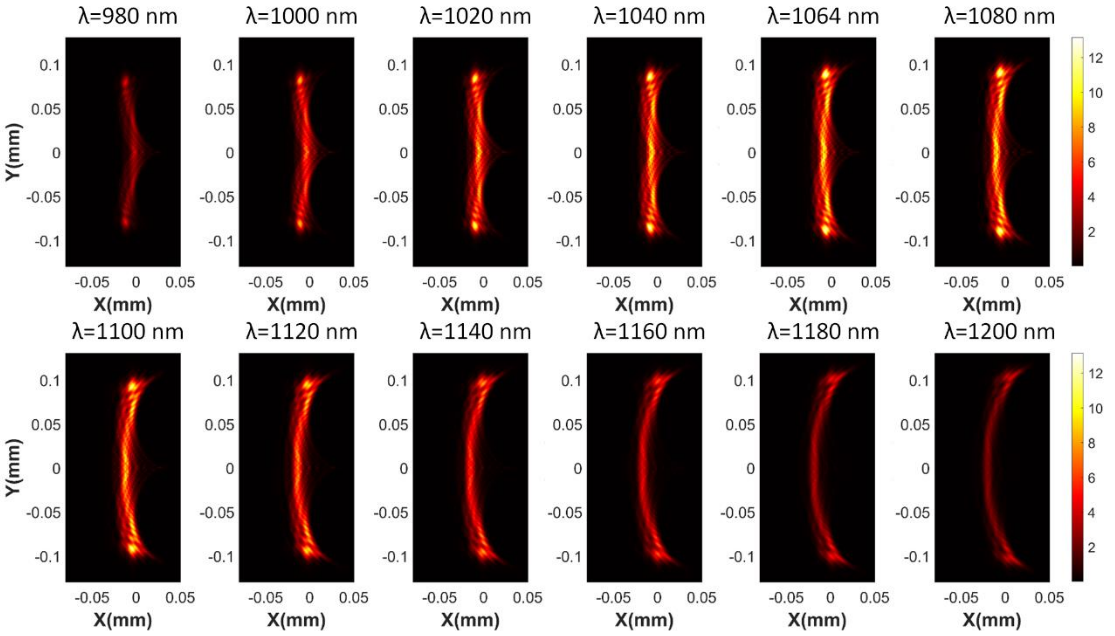

Figure S18. The log-polar coordinate transformed images using metasurfaces at different wavelengths

To overcome the chromatic aberration, we need to incorporate the wavelength dependence characteristics for the phase profile in Eq. S32. To this end, we can design the meta-atoms working at three different wavelengths (e.g. Red, Green and Blue), and design the phase profile $\varphi(x, y)$ for the three different wavelengths separately to avoid the chromatic aberration. As such we can obtain

$$\varphi(x, y, \lambda_R) = \frac{2\pi}{\lambda_R} \cdot \Phi(x, y), \tag{S34}$$

$$\varphi(x, y, \lambda_G) = \frac{2\pi}{\lambda_G} \cdot \Phi(x, y), \quad \text{(S35)}$$

$$\varphi(x, y, \lambda_B) = \frac{2\pi}{\lambda_B} \cdot \Phi(x, y), \quad \text{(S36)}$$

where λ_R, λ_G and λ_B are the wavelengths for Red, Green and Blue colors, respectively. By incorporating the phase profiles in Eqs. S34-S36 into metasurfaces, we can get aberration-free log-polar coordinate transformation at λ_R, λ_G and λ_B.

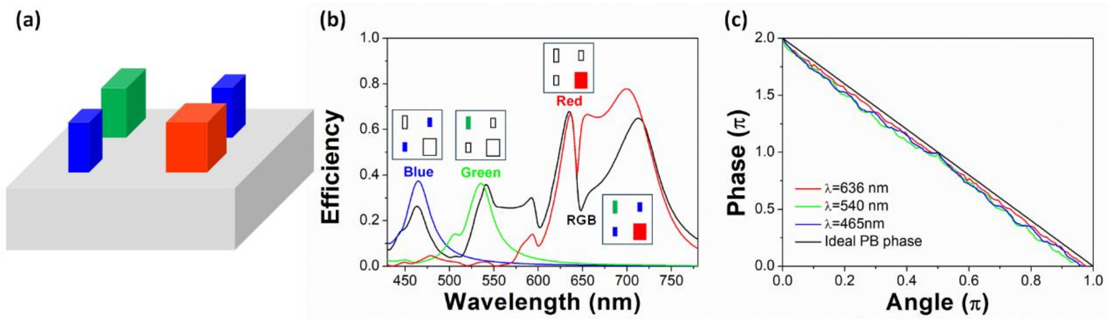

Figure S19. The design of meta-atoms for RGB log-polar coordinate transformation using metasurfaces. (a) The schematic of a meta-atom consisting of one silicon nanobar for Red, one silicon nanobar for Green, and two silicon nanobars for Blue. **(b)** The wavelength dependent right-to-left circular polarization conversion efficiency. Blue curve: only the Blue nanobars are enabled, Green curve: only the green nanobar is enabled; Red curve: only the Red nanobar is enabled; Black curve: all the four nanobars are enabled. (c) The PB phase as a function of rotation angle of the nanobars for λ_R=636 nm, λ_G=540 nm and λ_B=465 nm. The curve for the ideal PB phase is used for reference.

To verify our theoretical prediction, we designed a log-polar coordinate transformation metasurface operating for Red, Green and Blue colors (Fig. S19). We utilized crystalline silicon to build metasurfaces, whose absorption in visible spectrum is much smaller than amorphous silicon. To achieve image transformation for RGB colors, each meta-atom consists of four silicon nanobars with a height of 500 nm. For the Red nanobar in Fig. S19a, the length l_r=140 nm and the width w_r=110 nm, which gives a maximum right-to-left circular polarization conversion efficiency at λ_R=636 nm. Similarly, for the Green nanobar in Fig. S19a, the length l_g=110 nm and the width

w_g=40 nm, which yields a peak right-to-left circular polarization conversion efficiency at λ_G=540 nm. Due to the weak scattering efficiency at short wavelength, we arranged two nanobars for the phase control of blue light, and each Blue nanobar has a length of l_b=80 nm and a width of w_g=40 nm. The maximum right-to-left circular polarization conversion efficiency for the blue naobars is at λ_B=465 nm.

We then calculated the polarization conversion efficiency for the meta-atom consisting of those four nanobars. Compared with the efficiency for the meta-atoms with single color nanobars, the spectrum peaks are almost the same indicating weak cross-talk between nanobars. On the other hand, we utilized Pancharatnam-Berry (PB) phase method to control the phase imparted on the incident light. We calculated the imparted phase delay as a function of the rotation angle of nanobars. As can be seen in Fig. 19c, the phase delays for all the three wavelengths match the ideal PB phase very well.

To further evaluate the cross-talk between nanobars, we calculated the energy distribution for the meta-atom at λ_B=465 nm, λ_G=540 nm and λ_R=636 nm. As shown in Fig. S20, for each wavelength incidence, only the correlated nanobars have light field tightly confined with barely cross-talk between nanobars.

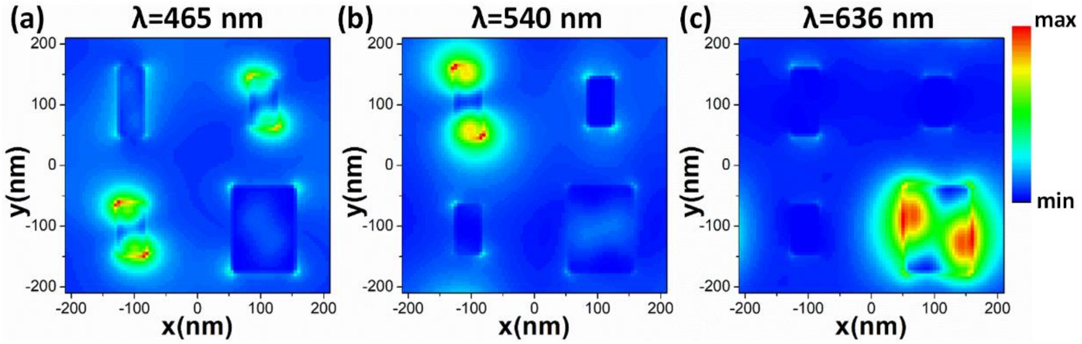

Figure S20. The spatial distribution of a meta-atom for $|E|^2$ at λ_B=465 nm, λ_G=540 nm and λ_R=636 nm. When the incident plane wave right circular polarized light wavelength is λ_B=465 nm, λ_G=540 nm and λ_R=636 nm, only the Blue, Green and Red nanobars are activated, respectively.

By using the nanobars in Fig. S19, we constructed a metasurface operating at λ_B=465 nm, λ_G=540 nm and λ_R=636 nm, and performed log-polar coordinate transformation for the ring-shaped image in Fig. 3a in the main text. Unlike the significant chromatic aberration for the metaurface designed with a single nanobar in Fig. S18, the transformed images using the RGB metasurface are almost the same for λ_B=465 nm, λ_G=540 nm and λ_R=636 nm (Fig. S21). Therefore, our metasurfaces can operate in the visible wavelength regime with at three RGB wavelengths.

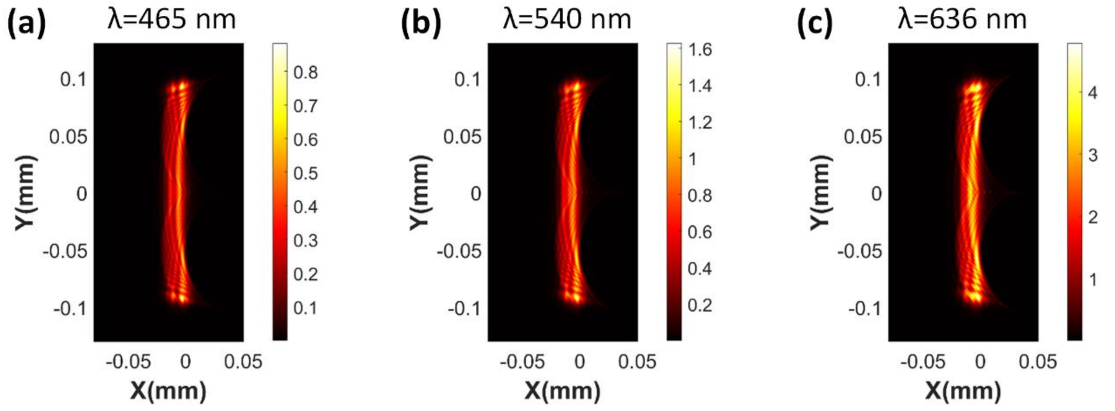

Figure S21. The log-polar coordinate transformed images using metasurfaces at different wavelengths in the visible spectrum.

11. The effect of linear translation on the Cartesian to log-polar coordinate transformation

For the Cartesian to log-polar coordinate transformation, we have the coordinate relations as below:

$$X(x, y) = a \cdot \ln \frac{r}{b}, \tag{S37}$$

$$Y(x, y) = -a \cdot \alpha, \tag{S38}$$

$$r = \sqrt{x^2 + y^2}, \tag{S39}$$

$$\alpha = \text{atan2}(y, x), \tag{S40}$$

Each input image has a unique transformed image which is solely determined by the coordinate transformation relationship in Eqs. S37-S40.

When the input image is scaled by $s = \frac{r'}{r}$ and rotated by $\theta = \alpha' - \alpha$, we have the expressions as below:

$$X'(x, y) = a \cdot \ln \frac{r'}{b}, \tag{S41}$$

$$Y'(x, y) = -a \cdot \alpha', \tag{S42}$$

Thus, we can obtain

$$X' = X + a \cdot \ln s, \tag{S43}$$

$$Y' = Y - a \cdot \theta, \tag{S44}$$

From Eqs. S43-S44, we know that the scale and rotation in Cartesian coordinate are transformed into linear translations along X and Y axes in the log-polar coordinate without changing the shape of the transformed image. We named such transformations as scale- and rotation- invariant transformations. As the scale and rotation operations determined by $s = \frac{r'}{r}$ and $\theta = \alpha' - \alpha$ are about the origin of Cartesian coordinate. scale- and rotation invariances are only valid when the input images are scaled and/or rotated about the origin of the Cartesian coordinate.

On the other hand, when the input image undergoes linear translation in the Cartesian coordinate (i.e. the center position of the input image is shifted to $r' = r - r_0$), we have

$$X'(x, y) = a \cdot \ln \frac{r - r_0}{b}, \tag{S45}$$

$$Y'(x, y) = -a \cdot \alpha, \tag{S46}$$

As such, we can get

$$X' = X + a \cdot \ln\left(1 - \frac{r_0}{r}\right), \tag{S47}$$

$$Y' = Y, \tag{S48}$$

From Eqs. S47-S48, we can see that the coordinate of the new transformed image along Y axis is still the same as the original input image. However, the coordinate of the new transformed image along X axis is translated by $a \cdot \ln\left(1 - \frac{r_0}{r}\right)$, which is not a constant. Therefore, in the case of linear translation of the input image in the Cartesian coordinate, the Cartesian to log-polar coordinate transformation is not invariance any more.

To comprehensively study the effect of the position of the input image on the shape of the transformed image in detail, we take the airplane shape demonstrated in Figs. 4-5 in the main text as an example. In this case, the geometric parameters of the airplane can be described by (*s*, *r*, *t*), in which *s*, *r* and *t* denote the scale, rotation angle and linear translation of the airplane. For the airplane shapes in Fig. 4 in the main text, the linear translation was zero (*t*=0). We re-plotted their Cartesian to log-polar coordinate transformations in Fig. S22. Obviously, the transformations were scale- and rotation-invariant when the linear translation was zero.

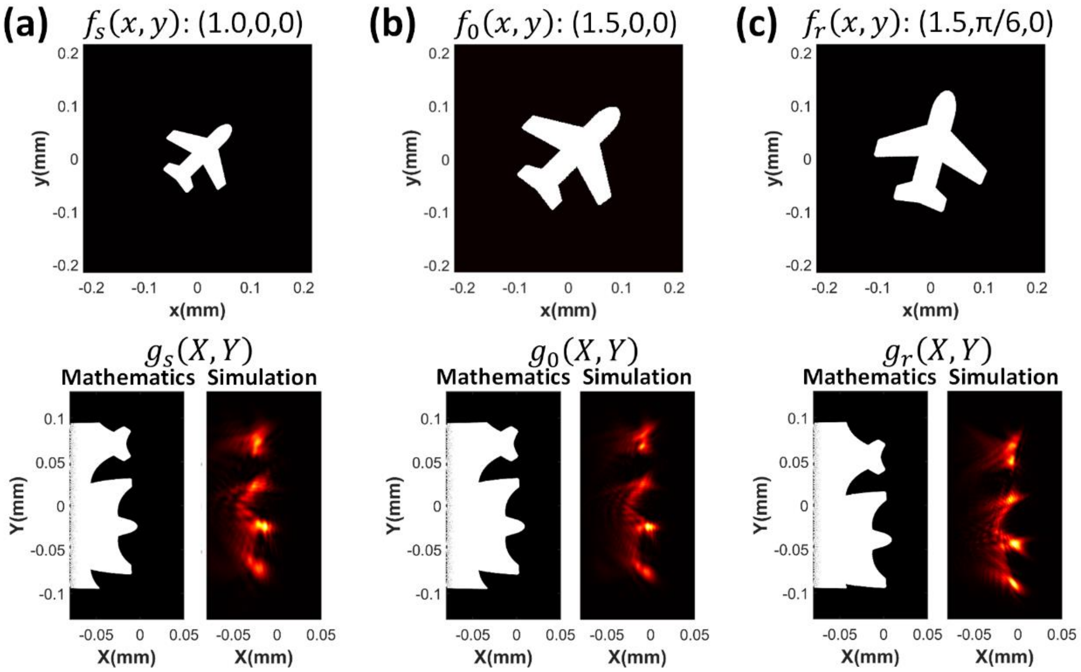

Fig. S22. The scale- and rotation- invariances of the log-polar coordinate transformation using metasurfaces. The log-polar coordinate transformation for input airplane shapes $f(x,y)$ in the Cartesian coordinate with different scale factors (s) and rotation angles (r) with zero linear translation (t=0). $f_s(x,y)$: s=1.0, r=0, t=0 (**a**); $f_0(x,y)$: s=1.5, r=0, t=0 (**b**); $f_r(x,y)$: s=1.5, r=π/6, t=0 (**c**). Top: the white and black regions for the input airplane shapes indicate the transmittance of 100% and 0%, respectively. Bottom: the mathematical and simulation transformed images in the log-polar coordinate for the input airplane shapes.

We then continued to study the effect of linear translation on the Cartesian to log-polar coordinate transformation. In this case, we chose $f_0(x,y)$ with a scaling factor of 1.5 and a rotation angle of 0 as an example. Figure S23a reveals that the auto-correlation for $g_0(X,Y)$ exhibited a bright spot at the origin, indicating a perfect match. As the linear translation along -X axis increased, the distortion of the transformed image became larger which can be verified by the wider spot in the cross-correlation maps. As such, we can conclude that the Cartesian to log-polar coordinate transformation is not translation-invariance as predicted in Eqs. S47-S48. Nevertheless, regarding the log-polar transformation of $f_1(x,y)$ with a linear translation of 21 μm, the cross-

correlation with $g_0(X,Y)$ still displayed a main bright spot in Fig. S23b. When the linear translation was larger than 21 μm, the cross-correlation maps showed broad bright regions, indicating poor similarities (Figs. S23c-S23e). Therefore, the tolerance for the center position of the airplane shapes is around 21 μm.

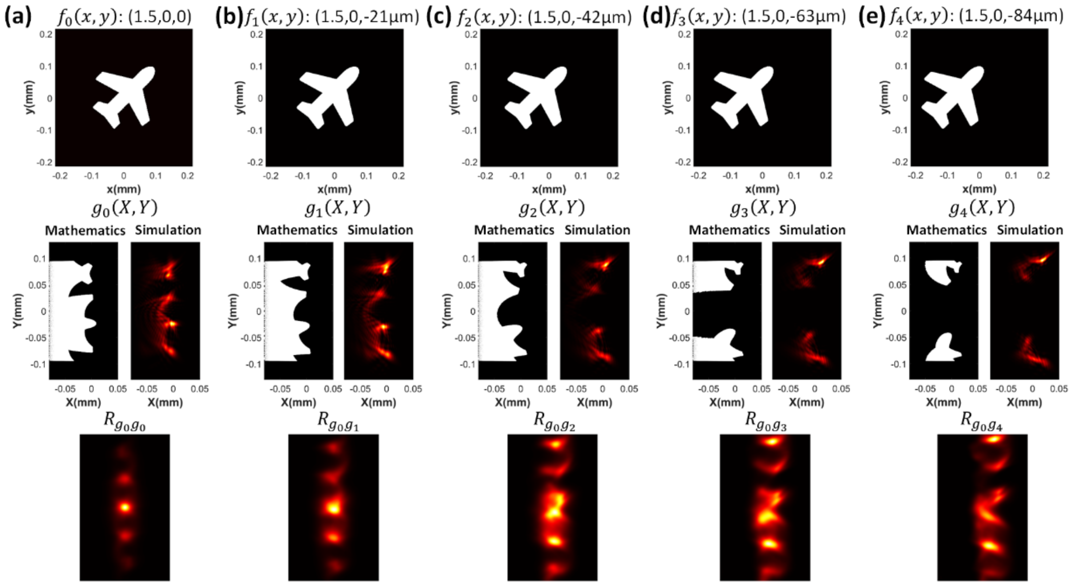

Fig. S23. The effect of linear translation on the Cartesian to log-polar coordinate transformation using metasurfaces. The log-polar coordinate transformation for input airplane shapes $f(x,y)$ in the Cartesian coordinate with different linear translations. $f_0(x,y)$: *s*=1.5, *r*=0, *t*=0 (**a**); $f_1(x,y)$: *s*=1.5, *r*=0, *t*=-21 μm (**b**); $f_2(x,y)$: *s*=1.5, *r*=0, *t*=-42 μm (**c**); $f_2(x,y)$: *s*=1.5, *r*=0, *t*=-63 μm (**d**) ; $f_2(x,y)$: *s*=1.5, *r*=0, *t*=-84 μm (**e**). Top: The white and black regions for the input airplane shapes indicate the transmittance of 100% and 0%, respectively. Middle: the mathematical and simulation transformed images in the log-polar coordinate for the input airplane shapes. Bottom: the correlations respect to $g_0(X,Y)$.

Having characterized the effect of linear translation on the Cartesian to log-polar coordinate transformation, we continued to study the scale-variances for the log-polar transformations of images with mismatched center positions. To this end, we compared $f_s(x,y)$ with $f_0(x,y)$, $f_1(x,y)$, $f_2(x,y)$, $f_3(x,y)$ and $f_4(x,y)$. For these airplane images, the scale factor for $f_s(x,y)$ was 1.0, while the scale factor for $f_0(x,y)$, $f_1(x,y)$, $f_2(x,y)$, $f_3(x,y)$ and $f_4(x,y)$ was 1.5. In addition to this, the linear

translation for $f_s(x,y)$ was zero, while the linear translations for $f_0(x,y)$, $f_1(x,y)$, $f_2(x,y)$, $f_3(x,y)$ and $f_4(x,y)$ were increased from 0 to -84 μm with a step size of -21 μm. It should be noted that the scaling of airplane images was respect to the center of the shape in this case. We then conducted the log-polar transformation for these airplane images using metasurfaces. After that, we calculated the correlations between $g_s(X,Y)$ and $g_s(X,Y)$, $g_0(X,Y)$, $g_1(X,Y)$, $g_2(X,Y)$, $g_3(X,Y)$, $g_4(X,Y)$, respectively. As shown in Fig. S24, the auto-correlation of $g_s(X,Y)$ displayed a bright spot in the origin, indicating a perfect match. The cross-correlation between $g_s(X,Y)$ and $g_0(X,Y)$ showed a shifted bright spot due to the scale-invariance of log-polar transformation. However, from $g_1(X,Y)$ to $g_4(X,Y)$, the cross-correlation respect to $g_s(X,Y)$ exhibited broadened bright regions, indicating poor similarities. Therefore, for airplane images with mismatched center positions, the Cartesian to log-polar coordinate transformation was not scaling invariant in the case of scaling of images about their own center positions.

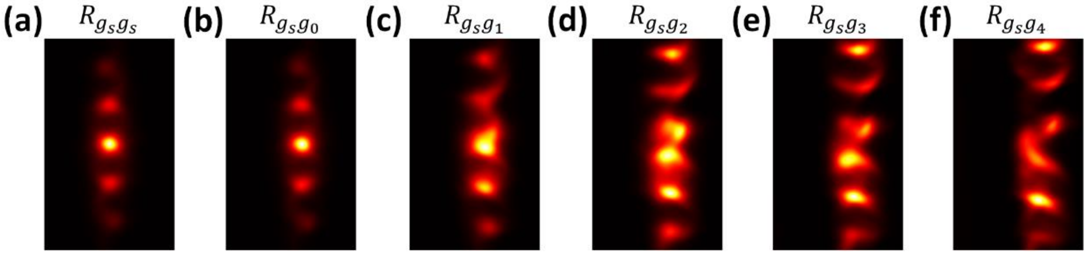

Fig. S24. The scale-variances for the log-polar transformations of images with mismatched center positions. The scaling of the images was about the center position of the airplane shape. The correlations between $g_s(X,Y)$ in Fig. S22 and $g_s(X,Y)$ **(a)**, $g_0(X,Y)$ **(b)**, $g_1(X,Y)$ **(c)**, $g_2(X,Y)$ **(d)**, $g_3(X,Y)$ **(e)**, $g_4(X,Y)$ **(f)** in Fig. S23.

Similarly, we also verified the rotation-variances for the log-polar transformations of images with mismatched center positions. For this purpose, we compared $f_r(x,y)$ with $f_0(x,y)$, $f_1(x,y)$, $f_2(x,y)$, $f_3(x,y)$ and $f_4(x,y)$. For these airplane images, the rotation angle for $f_r(x,y)$ was π/6, while the rotation angle for $f_0(x,y)$, $f_1(x,y)$, $f_2(x,y)$, $f_3(x,y)$ and $f_4(x,y)$ was 0. In addition to this, the linear translation for $f_r(x,y)$ was zero, while the linear translations for $f_0(x,y)$, $f_1(x,y)$, $f_2(x,y)$,

$f_3(x,y)$ and $f_4(x,y)$ were increased from 0 to -84 μm with a step size of -21 μm. It should be noted that the rotation of airplane images was respect to the center of the shape in this cacse. We then conducted the log-polar transformation for these airplane images using metasurfaces. After that, we calculated the correlations between $g_r(X,Y)$ and $g_r(X,Y)$, $g_0(X,Y)$, $g_1(X,Y)$, $g_2(X,Y)$, $g_3(X,Y)$, $g_4(X,Y)$, respectively. As shown in Fig. S25, the auto-correlation of $g_r(X,Y)$ displayed a bright spot in the origin, indicating a perfect match. The cross-correlation between $g_r(X,Y)$ and $g_0(X,Y)$ showed a shifted bright spot due to the rotation-invariance of log-polar transformation. However, from $g_1(X,Y)$ to $g_4(X,Y)$, the cross-correlation respect to $g_r(X,Y)$ exhibited broadened bright regions, indicating poor similarities. Therefore, for airplane images with mismatched center positions, the Cartesian to log-polar coordinate transformation was not rotation invariant in the case of rotation of images about their own center positions.

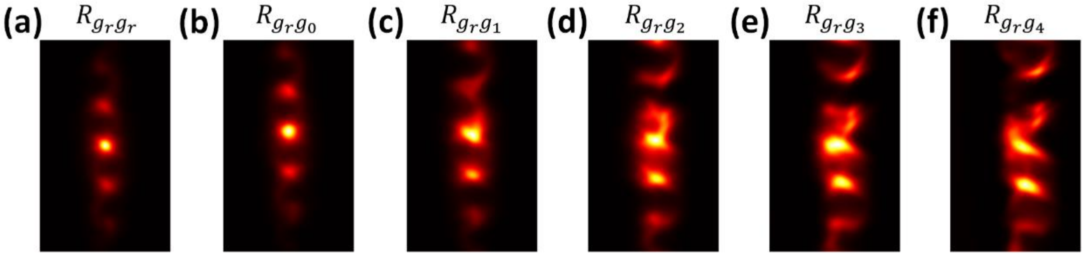

Fig. S25. The rotation-variances for the log-polar transformations of images with mismatched center positions. The rotation of the images was about the center position of the airplane shape. The correlations between $g_r(X,Y)$ in Fig. S22 and $g_r(X,Y)$ **(a)**, $g_0(X,Y)$ **(b)**, $g_1(X,Y)$ **(c)**, $g_2(X,Y)$ **(d)**, $g_3(X,Y)$ **(e)**, $g_4(X,Y)$ **(f)** in Fig. S23.

Nevertheless, regarding the log-polar transformation of $f_1(x,y)$ with a linear translation of 21 μm, the cross-correlations with $g_s(X,Y)$ and $g_r(X,Y)$ still displayed a single main bright spot in Figs. S24c and S25c, respectively. When the linear translation was larger than 21 μm, the cross-correlation maps showed broad bright regions, indicating poor similarities (Figs. S24d-S24f and S25d-S25f). Therefore, the tolerance for the center position of the airplane shapes is around 21 μm.

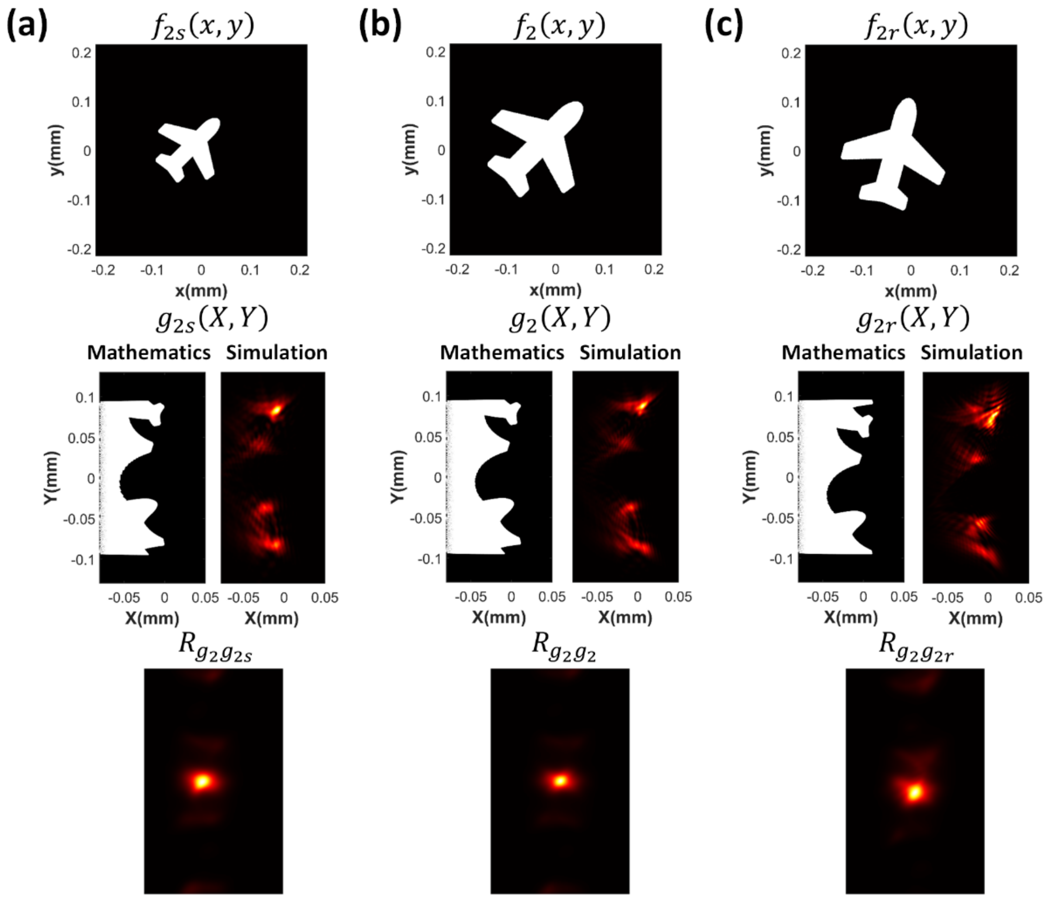

Fig. S26. The scale- and rotation- invariances for the log-polar transformations when the center positions of images were not at the origin of the Cartesian coordinate. Top: airplane shapes in the Cartesian coordinate with different scales and rotation angles. $f_{2s}(x,y)$: *s*=1.0, *r*=0 (**a**); $f_2(x,y)$: *s*=1.5, *r*=0 (**b**); $f_{2r}(x,y)$: *s*=1.5, *r*=π/6 (**c**). The white and black regions for the input airplane shapes indicate the transmittance of 100% and 0%, respectively. The scale and rotation of the images were about the origin of the Cartesian coordinate. Middle: the mathematical and simulation transformed images in the log-polar coordinate for the input airplane shapes. Bottom: the correlations respect to $g_2(X,Y)$.

We have demonstrated that the disappearance of scale- and rotation- invariance when the images have center position mismatches in the case of scale and rotation of images about their own center positions. We then studied the log-polar transformations for the images with scale and rotation operations with respect to the origin of the Cartesian coordinate when the center positions of images were not at the origin of the

Cartesian coordinate. With this in mind, we considered $f_2(x, y)$ whose center position was 42 μm away from the origin. After scaling and rotation transformation respect to the origin, we obtained $f_{2s}(x, y)$ and $f_{2r}(x, y)$ (Fig. S26). We then performed log-polar coordinate transformation for $f_2(x, y)$, $f_{2s}(x, y)$ and $f_{2r}(x, y)$, and we obtained $g_2(X, Y)$, $g_{2s}(X, Y)$, and $g_{2r}(X, Y)$. To characterize the similarity between $g_2(X, Y)$, $g_{2s}(X, Y)$, and $g_{2r}(X, Y)$, we conducted correlation analysis. As can be seen in Fig. S26, all the three correlation maps $R_{g_2 g_2}$, $R_{g_2 g_{2s}}$ and $R_{g_2 g_{2r}}$ displayed single narrow bright spot, indicating perfect matches. Therefore, if the scale and rotation of images were about the origin of the Cartesian coordinate, the log-polar transformations of images were still scale- and rotation- invariant when the center positions of images were not at the origin of the Cartesian coordinate.

12. Supplementary References

1. Hossack WJ, Darling AM, Dahdouh A. Coordinate Transformations with Multiple Computer-generated Optical Elements. *J Mod Opt* **34**, 1235-1250 (1987).

2. Aleksoff CC, Ellis KK, Neagle BD. Holographic Conversion of a Gaussian-Beam to a near-Field Uniform Beam. *Opt Eng* **30**, 537-543 (1991).
